# Modelling endogenous insulin concentration in type 2 diabetes during closed-loop insulin delivery

**DOI:** 10.1186/s12938-015-0009-5

**Published:** 2015-03-04

**Authors:** Yue Ruan, Hood Thabit, Malgorzata E Wilinska, Roman Hovorka

**Affiliations:** Department of Paediatrics, University of Cambridge, Cambridge, UK; Wellcome Trust-MRC Institute of Metabolic Science, University of Cambridge, Cambridge, UK

**Keywords:** Posthepatic endogenous insulin concentration, Closed-loop insulin delivery, Simulation, Modelling, Insulin secretion, Type 2 diabetes

## Abstract

**Background:**

Closed-loop insulin delivery is an emerging treatment for type 1 diabetes (T1D) evaluated clinically and using computer simulations during pre-clinical testing. Efforts to make closed-loop systems available to people with type 2 diabetes (T2D) calls for the development of a new type of simulators to accommodate differences between T1D and T2D. Presented here is the development of a model of posthepatic endogenous insulin concentration, a component omitted in T1D simulators but key for simulating T2D physiology.

**Methods:**

We evaluated six competing models to describe the time course of endogenous insulin concentration as a function of the plasma glucose concentration and time. The models were fitted to data collected in insulin-naive subjects with T2D who underwent two 24-h visits and were treated, in a random order, by either closed-loop insulin delivery or glucose-lowering oral agents. The model parameters were estimated using a Bayesian approach, as implemented in the WinBUGS software. Model selection criteria were used to identify the best model describing our clinical data.

**Results:**

The selected model successfully described endogenous insulin concentration over 24 h in both study periods and provided plausible parameter estimates. Model-derived results were in concordance with a clinical finding which revealed increased posthepatic endogenous insulin concentration during the control study period (*P* < 0.05). The modelling results indicated that the excess amount of insulin can be attributed to the glucose-independent effect as the glucose-dependent effect was similar between visits (*P* > 0.05).

**Conclusions:**

A model to describe endogenous insulin concentration in T2D including components of posthepatic glucose-dependent and glucose-independent insulin secretion was identified and validated. The model is suitable to be incorporated in a simulation environment for evaluating closed-loop insulin delivery in T2D.

**Electronic supplementary material:**

The online version of this article (doi:10.1186/s12938-015-0009-5) contains supplementary material, which is available to authorized users.

## Background

The ‘artificial pancreas’, or closed-loop insulin delivery system, is a state-of-the-art medical device designed to improve glycaemic control by automatically titrating insulin delivery according to real-time continuous glucose sensor values [1]. Over the past decade a large number of clinical studies have been undertaken to assess the safety and efficacy of the closed-loop system applied in adolescents [2], adults [3] and pregnant women [4] with type 1 diabetes (T1D). Home trials have also shown favourable results of improved glucose control in participants with T1D utilizing closed-loop during their normal daily life [5,6]. Further clinical investigations are in the pipeline aiming to extend the application of closed-loop to benefit a larger population with type 2 diabetes (T2D). Recently reported clinical study tested the feasibility and safety of closed-loop in insulin-naïve T2D subjects [7] and showed promising results.

Clinical studies in humans are costly and time consuming. A virtual testing environment where a population of synthetic subjects with diabetes is tested in a virtual computer space provides a plausible alternative to assess the closed-loop performance in a more time-saving and cost-effective way. It also allows to test scenarios which might not be ethical to perform in real human subjects, so that it gives insight into the limit and a better design of the underlying control algorithm [8,9]. The simulator usually incorporates, as a key component, a glucoregulatory mathematical model and a number of virtual subjects represented by individualized model parameters. However, most of the present simulation models have been developed for T1D [9]. With the growing interest of bringing closed-loop to people with T2D [10], a simulation environment with a glucoregulatory model reflecting glucose-insulin interactions in T2D is desired. In order to create a virtual population of subjects with T2D, a simulation model developed for T1D needs to be supplemented with a model of endogenous insulin secretion and ensuing endogenous insulin concentration.

With this objective in mind, we aimed to develop a posthepatic insulin concentration model and evaluate it with insulin/glucose data collected during a clinical trial in T2D subjects [7]. Six competing models were proposed and the best model was selected on the basis of our model selection criteria. The model of choice assumes a linear relationship between glucose-dependent posthepatic insulin secretion and plasma glucose, and incorporates two two-segment linear functions representing glucose-independent posthepatic insulin secretion after breakfast and lunch, respectively. The model successfully described endogenous insulin concentration data in 11 subjects with T2D, who underwent two 24-hour visits and were treated by either closed-loop insulin delivery or glucose-lowering oral agents.

## Methods

### Subjects and experimental design

We utilised data obtained from a clinical study involving 11 subjects [6 male, age 59.7 (12.1) years, BMI 30.1 (3.9) kg/m^2^, diabetes duration 8.0 (6.2) years and HbA1c 8.3 (0.8)%, mean (SD)] with non-insulin treated T2D, who underwent two 24-hour visits (from 9:00 a.m. on Day 1 until 9:00 a.m. on Day 2), four to six weeks apart, in a random order, treated by either closed-loop insulin delivery or glucose-lowering oral agents [7]. The study was approved by the Cambridge Research Ethics Committee and subjects signed informed consent prior to the commencement of the study.

During closed-loop visits, subjects’ routine diabetes therapy was discontinued the night before and replaced with model predictive control algorithm-driven subcutaneous insulin pump delivery of rapid-acting insulin analogue lispro (Eli Lilly, Indianapolis, IN), based on real-time continuous glucose monitoring. Meals were unannounced to the control algorithm and no prandial insulin bolus was given. During control visits, usual diabetes regimen was continued (metformin 92%, sulfonylureas 58%, DPP-4 inhibitors 33%). On both visits, subjects consumed matched 50-80 g carbohydrate meals at 0, 240 and 540 min relative to 9:00 a.m. on Day 1, and optional 15 g carbohydrate snacks. Blood samples were collected, every 15 minutes for measuring glucose concentration, and at the following time points: 0,15, 30, 45, 60, 90, 120, 150, 180, 210, 240, 255, 270, 285, 300, 330, 360, 390, 420, 450, 480, 510, 540, 555, 570, 585, 600, 630, 660, 690, 720, 750, 780, 810, 840, 870, 900, 960, 1020, 1080, 1140, 1200, 1260, 1320, 1380 and 1440 min, for measuring endogenous plasma insulin concentration.

### Insulin and glucose measurement

Samples were centrifuged immediately with plasma kept on ice and stored at – 80°C until assayed. Endogenous plasma insulin was measured by AutoDELFIA immunoassay (Perkin Elmer Life Sciences, Wallac Oy, Turku, Finland; inter-assay CV 3.1% at 29 pmol/l, 2.1% at 79.4 pmol/l, 1.9% at 277 pmol/l, 2.0% at 705 pmol/l) which has zero cross reactivity with insulin lispro administered during the closed-loop visits. Plasma glucose was measured by YSI2300STAT Plus Analyser (YSI, Fleet, Hampshire UK).

### Model description

**Figure 1:**
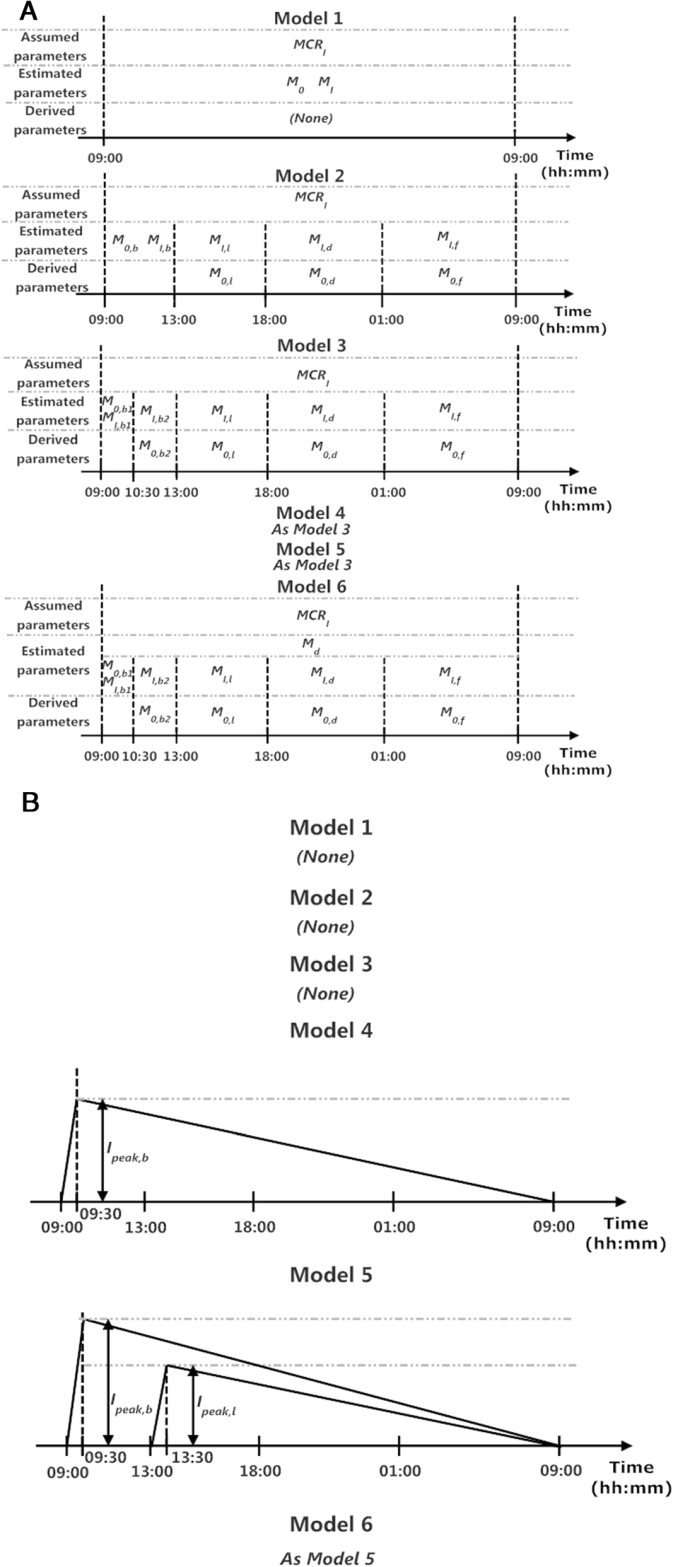
**Schematic representation of the six competing models.** The models are represented with the **(A)** glucose-dependent and **(B)** glucose-independent parameters.

We developed six models of increasing complexity to describe endogenous plasma insulin concentration [*I*_*ENDO*_(*t*)] as a function of plasma glucose concentration [*G*(*t*)] and time. A schematic representation of the six competing models is shown in Figure 1. We incorporate the glucose-dependent model components (in *Model 1* to *6*) and additionally the glucose-independent components (in *Model 4* to *6*). Models’ mathematical formulations are provided in Additional file 1.

*Model 1*, the base model, assumes a linear relationship between the rate of posthepatic insulin secretion *I*_*s*_(t) (mU/min) and measured plasma glucose concentration G(*t*) (mM). This assumption originates from previous studies which demonstrated linearity between the prehepatic insulin/C-peptide secretion and the plasma glucose concentration [11,12]. *I*_*ENDO*_(*t*) (mU/l), the measured endogenous plasma insulin concentration and model output, is assumed proportional to *I*_*s*_(t), through the inverse of the product of insulin metabolic clearance rate *MCR*_*I*_ (l/kg/min) and subject’s body weight *W* (kg). This assumption is based on the relatively short plasma insulin half-life (~5 min) compared to the sampling frequency in our study (15 to 60 min), allowing us to assume an instantaneous equilibration between posthepatic insulin appearance in plasma and plasma insulin [13]. The individual parameter values of *MCR*_*I*_ are taken from our previous study [13]. In Eq. (1), Additional file 1, G_b_ (mM) is the fasting plasma glucose concentration; *M*_*I*_ (mU/min/mM) is the posthepatic glucose sensitivity, representing the effect of unit change in blood glucose concentration on posthepatic insulin secretion and *M*_*0*_ (mU/min/mM) is the basal glucose sensitivity, representing effect of fasting plasma glucose on posthepatic insulin secretion.

*Model 2* has the same structure as *Model 1* but adopts different *M*_*I*_ and *M*_*0*_ values during postprandial and fasting periods. In this model, the 24-hour study period is divided into four time intervals, post-breakfast (4 h), post-lunch (5 h), post-dinner (7 h) and overnight (fasting time, 8 h), with each time interval holding a different *M*_*I*_ and *M*_*0*_ values (see Eq. (2), Additional file 1). In Eq. (2), Additional file 1, *M*_*I*_ turns into four parameters with subscripts *b*, *l*, *d* and *f*, representing the four time intervals. In the same fashion, *M*_*0*_ adopts four subscripts, with *M*_*0,b*_ being the estimated parameter from which values of *M*_*0,l*_, *M*_*0,d*_ and *M*_*0,f*_ can be derived. This derivation is to satisfy the assumption that the time course of *G*(*t*), *Is*(*t*) in a two-dimensional space is continuous throughout the study period. *t*_*l*_, *t*_*d*_, *t*_*f*_ and *t*_*end*_ are at 240, 540, 960 and 1440 min, respectively; these values are assigned according to meal times.

*Model 3* expands on *Model 2* by further dividing the post-breakfast period into two sub-periods with an additional breakpoint at *t*_*b*_ = 90 min, highlighting the difference of the first meal of the day after an overnight fast.

**Figure 2:**
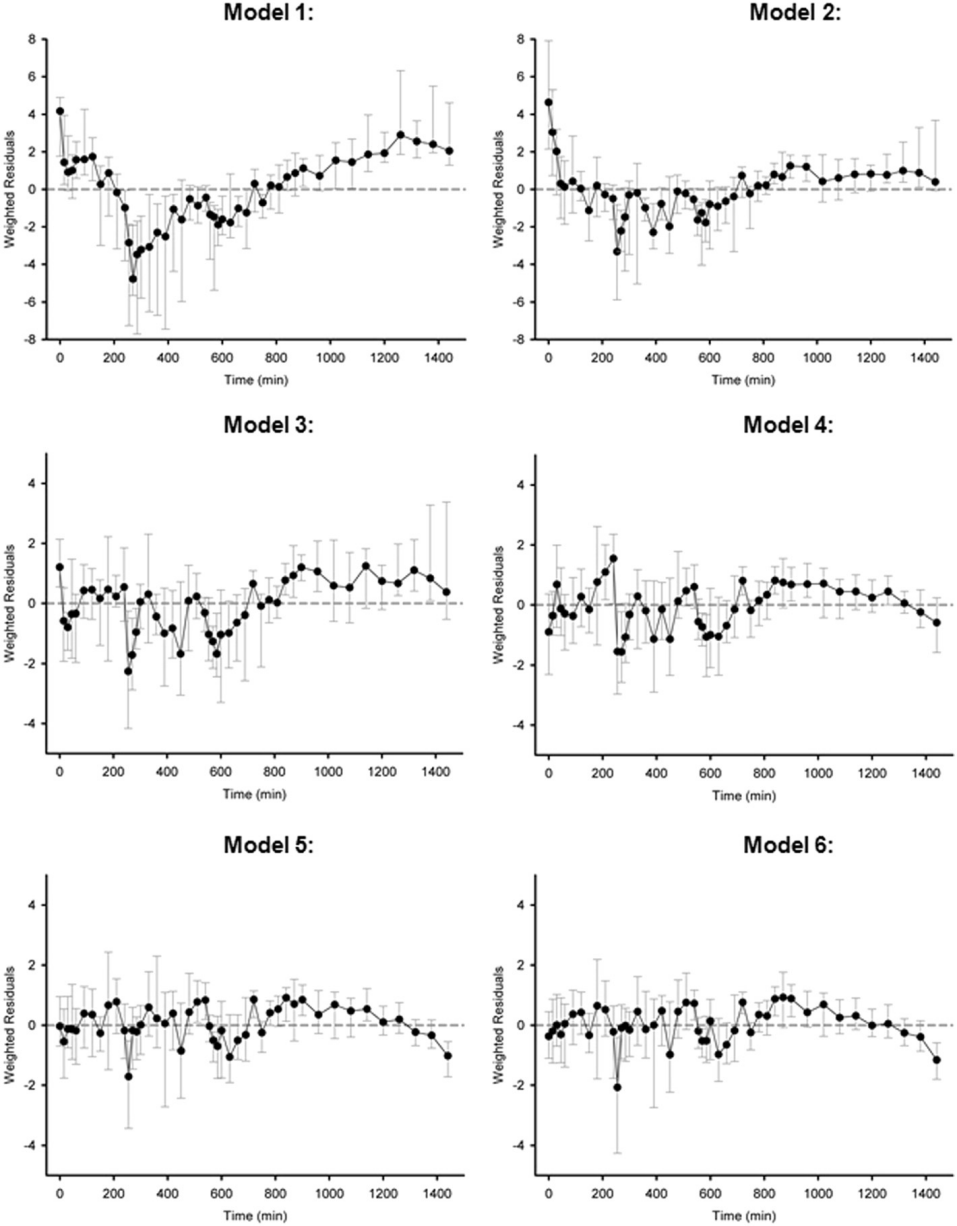
**Median weighted residuals obtained with the six competing models.** Weighted residuals are difference between model predictions and data divided by the standard deviation. The error bars represent the interquartile range (n = 11). Data collected from the closed-loop experiments were used.

The weighted residuals of *Model 3* (Figure 2) show postprandial underestimation of plasma insulin level indicating that underlying factors other than glucose stimulation influence the posthepatic insulin secretion. Thus, in *Model 4*, a two-segment piecewise linear function representing additional posthepatic insulin secretion after breakfast *I*_*add,b*_(*t*) is added. This additional flux assumes value 0 at *t* = 0, peaks at *t*=*t*_*peak,b*_ = 30 min and falls gradually to 0 at *t=t*_*end*_. The peak value *I*_*peak,b*_ (mU/min) is estimated.

The weighted residuals of *Model 4* (Figure 2) shows improved post-breakfast model fit but underestimation of insulin concentration still exist after lunch and dinner time. Therefore, in *Model 5*, another two-segment piece-wise linear function *I*_*add,l*_ (*t*) is added after lunch time. Similar to the post-breakfast flux in *Model 3*, the post-lunch flux assumes value 0 at *t* = *t*_*l*_ = 240 min, peaks at *t=t*_*peak,l*_ =270 min and drops to 0 at *t=t*_*end*_. The peak value *I*_*peak,l*_ (mU/min) is estimated.

Finally, in addition to *Model 5*, *Model 6* assumes a derivative action of *G*(*t*)on the posthepatic insulin secretion as a number of studies claimed derivative action of glucose concentration on prehepatic insulin secretion [11]. *Id*(*t*) (mU/min) is the posthepatic insulin secretion triggered by the positive rate of change of glucose concentration; *M*_*d*_ (mU/mM) is the dynamic glucose responsivity representing the ability of glucose rate of change to stimulate posthepatic insulin secretion (Eq. (6), Additional file 1).

### Model selection and parameter estimation

Parameters of the six competing models were identified on clinical data obtained from the closed loop periods and the results were used in model selection criteria. The parameters of the model of choice were subsequently identified on data obtained from the control period.

In the first instance, all six models were numerically identified using glucose/insulin data obtained from the closed-loop period, using a Bayesian approach. WinBUGS (Bayesian inference Using Gibbs Sampling for Windows) software version 1.4 (MRC Biostatistics Unit, Cambridge, UK) [14] was used for model identification. The measurement error in *I*_*ENDO*_(*t*) was assumed to be uncorrelated, normally distributed with zero mean, and with a coefficient of variation at 6%. The plasma glucose concentration represented by *G*(*t*), being the model input, was assumed error free.

The initial model identification results were used to select the best model on the basis of the following criteria: (i) the deviance information criterion (*DIC*) which is a measurement of model parsimony, taking into account the goodness of fit and the model complexity [15], (ii) the weighted residual profiles documenting overall model ability to describe the data, and (iii) the presence of zero estimated parameters which indicate increased model complexity leading to over-fitting. The model of choice was then identified on data obtained from the eleven control periods.

The individual parameter sets of the best model were identified using the Bayes Theorem:

$$ p\left(\theta \Big|y\right)=\frac{p\left(\theta \right)p\left(y\Big|\theta \right)}{{\displaystyle \int p\left(\theta \right)p\left(y\Big|\theta \right)d\theta }}\propto p\left(\theta \right)p\left(y\Big|\theta \right) $$

where *y* is the dataset of the posthepatic endogenous insulin concentration measurements, *θ* represents the unknown parameters *M*_*I,b1*_*, M*_*I,b2*_*, M*_*I,l*_*, M*_*I,d*_*, M*_*I,f*_*, M*_*0,b1*_*, M*_*0,b2*_*, M*_*0,l*_*, M*_*0,d*_*, M*_*0,f*_*, I*_*peak,b*_ and *I*_*peak,l*_. The prior information on the model parameters is represented by their a priori distributions *p*(*θ*)which are updated by the likelihood *p*(*y*|*θ*), yielding parameters’ a posteriori distributions *p*(*θ*|*y*). The integral ∫*p*(*θ*)*p*(*y*|*θ*)*dθ* acts as a scale factor.

In the present study, vague prior information was assigned to each parameter. For each study period, it took WinBUGS 550 seconds to run 100,000 iterations and produce posterior parameter distributions. A standard 64-bit desktop PC (OPTIPLEXTM 7010, DELLTM Computers Ltd) was used. The first 10,000 iterations were discarded as burn-ins to allow the initial stabilization of the Markov chain [16].

### Statistical analysis

Results are shown as median (interquartile range), if not differently indicated. The Wilcoxon signed-rank test was used to assess the between-sample difference due to the non-normal data. SPSS software version 21 [17] was used to carry out the statistical analysis where *P* values less than 0.05 were considered statistically significant.

## Results

### Endogenous plasma insulin concentration

Table 1 compares the area-under-curve (*AUC*_*ins*_) of measured endogenous plasma insulin concentration between visits. A significant increase in the control period *AUC*_*ins*_ can be observed during the three postprandial time intervals as well as during the fasting conditions (*P* < 0.05) suggesting an increase in posthepatic insulin secretion throughout the control period.

**Table 1:** **The area under the curve (** ***AUC***_***ins***_ **) of the model-derived endogenous plasma insulin concentration**

	**Closed-loop (n = 11)**	**Control (n = 11)**	***P*** **-value**
	***AUC***_***ins***_ **(10** ^**3**^ **· min · mU/l)**		
Post-breakfast	6.4 (3.6,7.9)	7.4 (4.9,10.4)	0.006
Post-lunch	7.4 (4.0,10.0)	10.9 (8.2,13.9)	0.026
Post-dinner	6.7 (4.3,10.5)	7.8 (6.9,15.9)	0.048
Fasting	3.1 (2.1,3.8)	4.6 (3.2,7.7)	0.003

### Model selection

Figure 2 illustrates weighted residual profiles obtained with the six competing models. *Model 5* and *Model 6* result in comparable profiles and provide the best fit to the data. Table 2 shows results of the *DIC* analysis. As expected, the deviance $\overline{D}$ decreased and the ‘effective number of parameters’ *p*_*D*_ increased with the increasing model complexity. *Model 6* outperforms the other five models in terms of the lowest *DIC* value. However, following model identification, we noted that the parameter *M*_*d*_, exclusive to *Model 6*, was estimated to be zero in 9 out of the 11 tested data sets. *Model 6* was therefore rejected and *Model 5* became the model of choice as it adequately described the data and held the second lowest, after Model 6, *DIC* value.

**Table 2:** **Results of DIC analysis for the six competing models**

**Model**	$\overline{D}$	$\widehat{D}$	***p***_***D***_	***DIC***	***d***
1	9003	8982	21	9024	5597
2	6435	6409	26	6461	3034
3	4482	4435	47	4529	1102
4	4079	4024	55	4134	707
5	3394	3335	59	3453	26
6	3357	3287	70	3427	0

$\overline{D}=$ posterior mean of -2log(likelihood); $\widehat{D}=$ 2log(likelihood) at posterior mean of stochastic nodes; ${p}_D=\kern0.5em \overline{D}-\widehat{D}$, defines “effective number of parameters”; $DIC=\overline{D} + {p}_D$, is deviance information criteria. *d* is the difference between the present model’s *DIC* and the lowest *DIC*.

### Model-derived results

**Figure 3:**
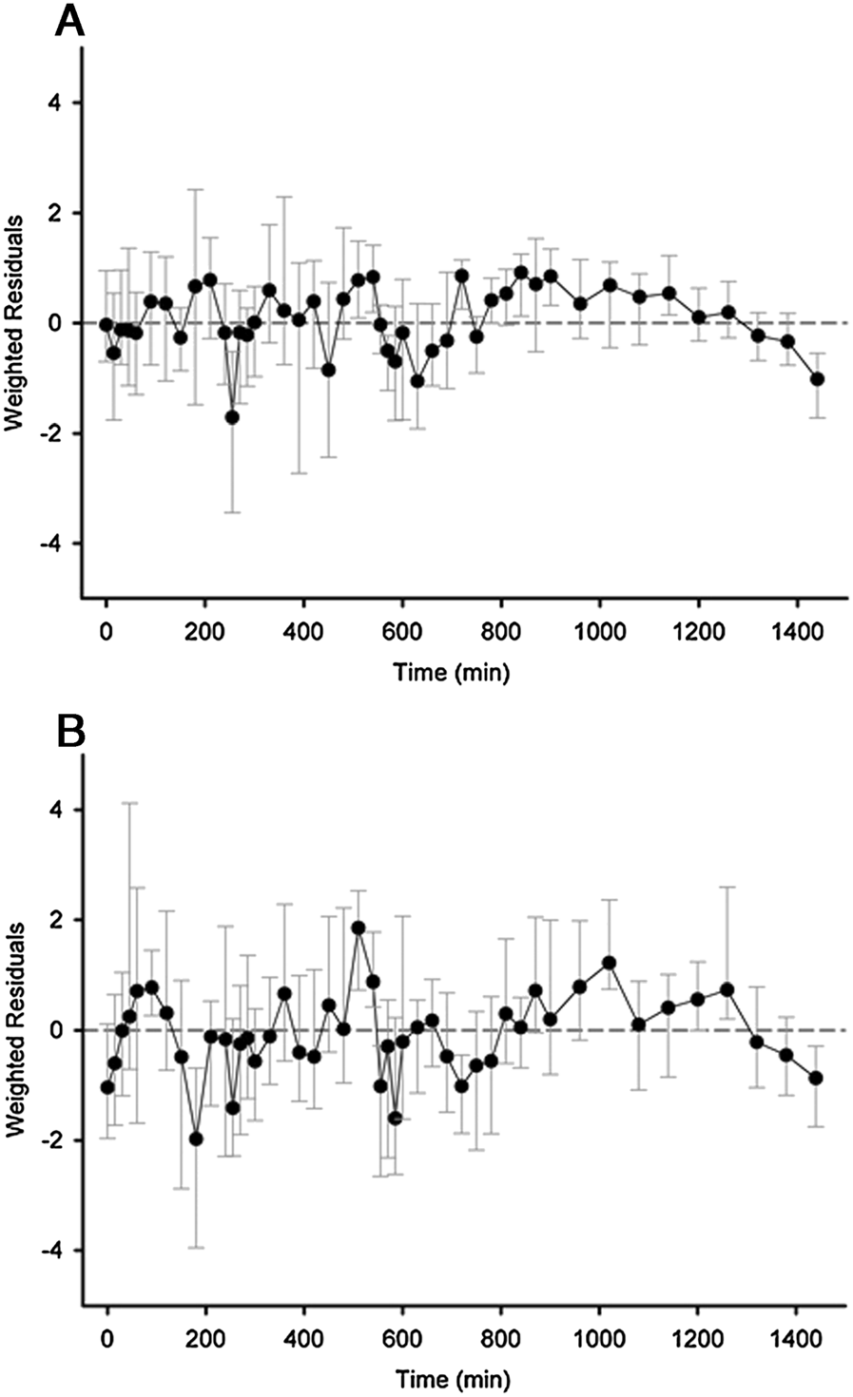
**Weighted residuals during (top panel A) closed-loop and (bottom panel B) control period using Model 5.**

**Figure 4:**
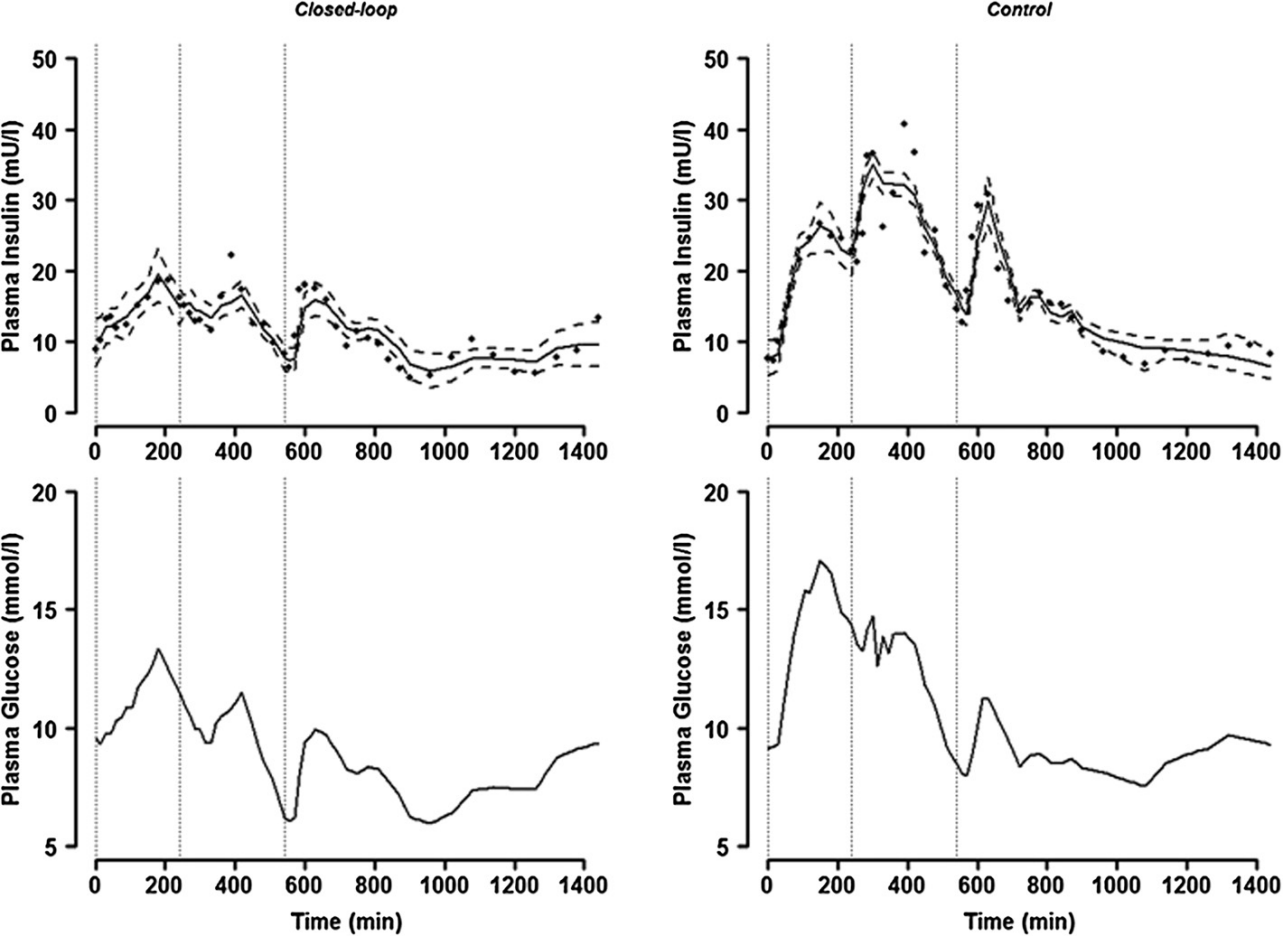
**Sample model fit obtained with subject 7.** The model fit (with *Model 5*) to endogenous plasma insulin concentration (upper panel) with plasma glucose excursion (lower panel) during closed-loop (left panel) and control period (right panel); solid line represents model prediction, dashed line 95% intervals, the dotted vertical lines indicate meal time and full circles dots represent measurements).

Figure 3 shows weighted residual profiles for both closed-loop and control periods documenting overall plausible model fit to data using *Model 5*. Individual model fits are provided in Additional file 1. An example fit shown in Figure 4 confirms satisfactory model behaviour during both study periods and a positive correlation between plasma glucose and endogenous plasma insulin levels.

Table 3 reports estimates of model parameters *M*_*I*_, *M*_*0*_ and *I*_*peak*_. Median estimates of *M*_*I,f*_ are zero on both occasions. When comparing the parameter estimates between visits, there is no significant difference in either *M*_*I*_ or *M*_*0*_ (*P* > 0.05) – the ability of glucose concentration to stimulate posthepatic insulin secretion is similar between treatments. However, *I*_*peak,l*_ holds a higher median estimate during the control period compared to the closed-loop visit (*P* < 0.05).

**Table 3:** **Parameter estimates for** ***Model 5***

**Model parameters**	**Closed-loop (n = 11)**	**Control (n = 11)**	***P*** **-value**
*M*_*I,b1*_ (mU/min/mM)	2.6 (1.6,3.2)	4.3 (2.0,5.3)	0.11
*M*_*I,b2*_ (mU/min/mM)	1.0 (−0.2,2.7)	1.6 (0.1,4.7)	0.59
*M*_*I,l*_ (mU/min/mM)	5.0 (1.8,6.6)	2.7 (0.0,8.5)	0.48
*M*_*I,d*_ (mU/min/mM)	3.0 (2.5,4.8)	4.8 (0.2,8.2)	0.25
*M*_*I,f*_ (mU/min/mM)	0.0 (0.0,1.8)	0.0 (0.0,6.0)	0.59
*M*_*0,b1*_ (mU/min/mM)	1.1 (0.9,1.7)	1.3 (0.3,1.5)	0.48
*M*_*0,b2*_ (mU/min/mM)^*^	1.9 (1.6,2.2)	1.7 (1.0,2.7)	0.72
*M*_*0,l*_ (mU/min/mM)^*^	1.8 (1.1,2.8)	1.8 (1.3,2.7)	0.66
*M*_*0,d*_ (mU/min/mM)^*^	1.6 (0.9,2.0)	1.6 (1.0,2.3)	0.59
*M*_*0,f*_ (mU/min/mM)^*^	0.7 (0.4,1.1)	0.8 (0.6,1.5)	0.48
*I*_*peak,b*_ (mU/min)	7.8 (0.0,14.0)	17.8 (0.0,21.1)	0.27
*I*_*peak,l*_ (mU/min)	0.0 (0.0,13.9)	13.2 (0.0,30.5)	0.02
*MCR*_*I*_ (l/kg/min)^**^	0.013 (0.005)	as Closed-loop	NA

^*^Derived parameter.

^**^Assumed parameter, mean (SD).

Table 4 compares model-derived *AUC*_*ins*_ and its glucose-dependent and glucose-independent components between visits. The *AUC*_*ins*_ is significantly larger during the control period (*P* < 0.05), with the excess amount only attributable to the glucose-independent component (*P* < 0.05). The glucose-dependent *AUC*_*ins*_ show no discordance between visits (*P* > 0.05).

**Table 4:** **Comparison of model derived glucose-dependent, glucose–independent plasma insulin concentration and the total** ***AUC***_***ins***_

	**Closed-loop (n = 11)**	**Control (n = 11)**	***P*** **-value**
	***AUC***_***ins***_ **(10** ^**3**^ **· min · mU/l)**		
Glucose-dependent	14.8 (12.1,21.0)	15.1 (11.3,19.5)	0.930
Glucose-independent	9.8 (3.7,14.9)	21.3 (8.5,24.5)	0.004
Total	23.5 (15.3,30.0)	35.8 (24.7,49.7)	0.006

## Discussion

We present a model describing the time course of 24-h endogenous plasma insulin concentration in people with T2D. The model was identified on insulin/glucose data obtained from 11 insulin-naïve T2D subjects who underwent two 24-hour visits treated by either closed-loop insulin delivery or glucose-lowering oral agents. Both clinical and model-derived results suggested an increase in the amount of posthepatic endogenous insulin secretion during the control period, namely the oral agents-treated period. The modelling results further suggested that the surplus insulin secretion was glucose-independent. We consider the model suitable to be incorporated in a simulation environment for testing closed-loop inulin delivery performance in T2D.

In the model development process, six competing models of increasing complexity have been evaluated. All models adopted the following assumptions: a linear relationship exists between plasma glucose concentration and posthepatic insulin secretion rate; *MCR*_*I*_ is identical between study visits; the equilibration is instantaneous between posthepatic insulin appearance in plasma and plasma insulin, reflecting a short plasma insulin half-life relative to the frequency of plasma insulin measurements. The latter assumption was adopted in our previous study during which individual *MCR*_*I*_ values were estimated [13].

In order to select the model best representing our data sets, three criteria were adopted. Based on the DIC criterion (Table 1), *Model 6* would have been chosen as the most parsimonious. However, the parameter estimates of *M*_*d*_ converged to zero in 82% of the cases implying that *Model 6* collapsed into *Model 5* and that the contribution of the derivative action of glucose concentration on posthepatic insulin secretion was overall negligible. Therefore, *Model 6* was rejected and *Model 5* became our model of choice. In terms of model parameter estimation, we found that, unlike *M*_*I,b*_, *M*_*I,l*_ and *M*_*I,d*_, which represent posthepatic glucose sensitivities after breakfast, lunch and dinner and which had non-zero estimates, the median estimates of *M*_*I,f*_ were zero for both visits. This may suggest that the linear relationship between plasma glucose and plasma insulin may be less pronounced during the fasting period, probably due to the less variable glucose and insulin levels, compared to postprandial periods. A more sophisticated model structure may be required to describe the ‘plasma glucose-endogenous plasma insulin’ relationship overnight.

Additional file 1 shows plots of measured plasma glucose concentration versus endogenous plasma insulin concentration. Given the assumption of linearity, we should have observed a unique linear dependency between measurements for each of the time intervals. However, in a few subjects, namely in subjects 2, 3, 4, 6, 8 during the closed-loop period and subjects 2, 4, 6, 8, 10 during the control period, the endogenous plasma insulin concentration remains high or continues to increase despite a pronounced drop in the plasma glucose level, especially after the first meal of the day. This observation encouraged us to incorporate the glucose-independent insulin secretion component.

The bioactive incretins, namely glucagon-like peptide-1 (GLP-1) and glucose-dependent insulinotropic polypeptide (GIP), are hormones released from the small intestine in response to oral meal intake, acting as enhancers to endogenous insulin secretion. Woerle et al. demonstrated that incretins play a relatively more important role in amplifying insulin secretion in people with T2D compared to healthy subjects [18]. We therefore hypothesize that the glucose-independent insulin secretion functions in our model may account for, at least in part, the insulinotropic effect of incretin hormones, and may correspond to the ‘potentiation’ of insulin secretion after meal proposed by Mari et al. [11].

The excess amount of glucose-independent insulin secretion during the control period revealed by the modelling process may be attributed to the effect of glucose-lowering agents. As suggested in literature, metformin (taken by 92% of the studied subjects during the control period) enhances GLP-1 action [19], sulfonylureas (taken by 58% of the studied subjects) stimulates endogenous insulin secretion [20] and DPP-4 inhibitors (taken by 33% of the studied subjects) improves insulin secretion and prolongs GLP-1 action [21].

Insulin and C-peptide are co-secreted equimolarly by the pancreatic beta-cells. After travelling through the liver, about 50% of insulin is extracted before entering the plasma while C-peptide is not affected. The prehepatic insulin secretion profile can, therefore, be reconstructed using a model of C-peptide kinetics [22] where the beta-cell function can be assessed quantitatively [11,12,23]. However, the objective of this study was to describe posthepatic insulin concentration with a relatively simple model. Thus, plasma C-peptide concentration was not measured in the present study.

The model can be implemented in a simulation environment designed specifically for accelerating the development of closed-loop insulin delivery systems for T2D. The endogenous insulin secretion characteristics of the virtual subjects with T2D will be represented by individual model parameters identified in the present study. The model may also be used in pharmaceutical development. In a possible application, model parameters identified using data collected before and after the ingestion of a tested glucose-lowering agent could be compared. The comparison may help to evaluate the efficacy of a new drug to influence, for instance, the posthepatic glucose sensitivity *M*_*I*_. The model’s ability to quantitatively evaluate effects of altered glucose turnover on endogenous insulin secretion, when patients’ usual glucose-lowering oral agent is replaced with subcutaneous insulin therapy, may also be of clinical relevance [24].

A possible limitation of the present study is the assumption of *MCR*_*I*_ consistency between study visits. However, no studies to date have shown that discontinuing exogenous insulin administration or oral hypoglycaemic therapy could affect plasma insulin clearance rate. Our previous study in subjects with T1D [25] supported this assumption by demonstrating reproducibility of all insulin aspart pharmacokinetic parameters including the insulin clearance rate.

In conclusion, a model describing 24-h posthepatic endogenous insulin concentration in T2D is proposed. The model was evaluated on clinical data obtained in subjects with T2D treated by either closed-loop insulin delivery (closed loop period) or by glucose-lowering oral agents (control period). The clinical results showed an increased posthepatic insulin secretion during the control period. The modelling results further suggested that this excess is glucose-independent. The presented model is suitable for application in an *in silico* simulation environment for testing closed-loop insulin delivery algorithms in T2D.

## Additional file

Additional file 1:
**Mathematical formulation of the six competing models.** Model fits to the individual data sets. Plasma glucose concentration vs. endogenous plasma insulin concentration.
