# Supplementary material for: Modelling endogenous insulin concentration in type 2 diabetes during closed-loop insulin delivery
Source: Biomed Eng Online. 2015 Mar 4;14:19. doi: 10.1186/s12938-015-0009-5 (PMC4359432; doi:10.1186/s12938-015-0009-5)
Supplement: Additional file 1: — Mathematical formulation of the six competing models. Model fits to the individual data sets. Plasma glucose concentration vs. endogenous plasma insulin concentration. [file 12938_2015_9_MOESM1_ESM.docx]

Additional file 1. Mathematical formulation of the six competing models

Model 1:

| __ | (1) |
| --- | --- |

where I_ENDO_(t) is the measured endogenous plasma insulin concentration (mU/l); I_s_(t) is the posthepatic insulin secretion rate (mU/min); MCR_I_ is the insulin metabolic clearance rate (l/kg/min); W is subject’s body weight (kg); G(t) is the measured plasma glucose concentration (mM); G_b_ is the fasting plasma glucose concentration (mM); M_I_ is the posthepatic glucose sensitivity, representing the effect of unit change in blood glucose concentration on posthepatic insulin secretion (mU/min/mM), M_0_ is the basal glucose sensitivity, representing effect of fasting plasma glucose on posthepatic insulin secretion (mU/min/mM).

Model 2:

| __ | (2) |
| --- | --- |

where M_I_ and M_0_ turn into four parameters respectively with subscripts b, l, d and f representing four different time intervals; t_l_, t_d_, t_f_ and t_end_ are time breakpoints at 240, 540, 960 and 1440 min, respectively.

Model 3:

| __ | (3) |
| --- | --- |

where M_I,b_ turns into M_I,b1_ and M_I,b2_ with additional breakpoint t_b_ = 90 min.

Model 4:

| __ | (4) |
| --- | --- |

where I_add,b_(t), a two-segment piecewise linear function representing additional posthepatic insulin secretion after breakfast is added (mU/min), which arises from 0 at t = 0, peaks at t = t_peak,b_ = 30 min and falls gradually to 0 at t = t_end_.

Model 5:

| __ | (5) |
| --- | --- |

where I_add,l_(t), a second piece-wise linear function is added after lunch time (mU/min), which arises from 0 at t = t_l_ = 240 min, peaks at t = t_peak,l_ =270 min and drops to 0 at t = t_end_.

Model 6:

| __ | (6) |
| --- | --- |

where I_d_(t) is the posthepatic insulin secretion triggered by the positive rate of change of glucose concentration (mU/min); M_d_ is the dynamic glucose responsivity representing the ability of glucose rate of change to stimulate posthepatic insulin secretion (mU/mM).

Model fits to the individual data sets.

Figure A1. Model fits to endogenous plasma insulin concentration during closed-loop (A) and control periods (B) (solid line: model prediction, dashed line: 95% credible intervals, dotted line: meal time, points: measurements).

**A**

**B**

Plasma glucose concentration vs. endogenous plasma insulin concentration.

Figure A2. Trajectories of plasma glucose vs. endogenous plasma insulin during closed-loop (A) and control periods (B).

A

**B**
